# The characteristics of positive and confusing hand X-ray signs in diagnosing Kashin–Beck Disease in children in China

**DOI:** 10.1038/s41598-018-21675-5

**Published:** 2018-02-19

**Authors:** Quan-Quan Song, Hui Liu, Wei Lian, Yue Wang, Li-Yan Sun, Mei Gao, Yun-Qi Liu, Hong-Xia Deng, Qing Deng, Ning Liu, Jun Yu

**Affiliations:** 1 Institute for Kaschin-Beck Disease Control and Prevention, Chinese Center for Disease Control and Prevention, Harbin Medical University, Harbin, 150081 Heilongjiang China; 2 Key Laboratory of Etiology and Epidemiology, National Health and Family Planning Commission, Beijing, 23618504 China

## Abstract

When screening for Kashin–Beck disease (KBD) in children, hand X-ray examination is the most important measure. However, there is high rate of misdiagnosis because of confusing X-ray signs. We studied the characteristics of positive and confusing hand X-ray signs. Clinical and radiological examinations were conducted in all 7- to 12-year-olds in selected villages from some KBD and non-KBD areas. We analysed the radiological and epidemiological characteristics of the X-ray signs of KBD and the confusing signs. Images from 3,193 children were valid. No cases of KBD were found. Seventeen children (0.53%) had X-ray signs positive for KBD. The confusing X-ray signs included closure reaction of metaphysis-epiphysis (CRME, 14.28%), thumb variation (0.22%), little finger variation (8.89%), the second metacarpal-phalangeal variation (0.13%) and cystic change (3.85%). The onset of CRME in children occurred earlier in girls (9) than in boys (10). The onset occurred earlier in KBD areas (9) than in non-KBD areas (10). The onset occurred earlier in Han children (9) than in Tibetan children (11). In summary, KBD was effectively controlled in all investigated KBD endemic villages, and the age range should be adjusted to 7- to 11-year-olds in Han children to reduce the misdiagnosis rates in KBD surveillance.

## Introduction

Kashin–Beck disease (KBD) is a serious endemic osteoarthrosis in China. It primarily occurs in children and adolescents during bone-joint development, resulting in multiple articular cartilage, symmetrical degeneration, and necrosis; moreover, the disease is accompanied by repeated proliferation and repair phenomena^1^. KBD begins in metaphyses, and then, it spreads to epiphyses and diaphyses, especially the middle segments and basal segments of the index, middle and ring fingers.

Currently, KBD has been effectively controlled in China through the implementation of comprehensive preventive measures. The numbers of new clinical KBD cases has been steadily decreasing. However, due to the unclear aetiology^2^, KBD remains a substantial threat to 22 million people living in KBD endemic areas in China. Therefore, effective monitoring remains necessary. Hand X-rays in children has been the most important KBD screening method^3–6^. A previous study defined six positive X-ray changes in KBD based on the location in the child’s hand: metaphysis type (I), distal end phalanx type (II), metaphysis-epiphysis type (III) metaphysis distal end phalanx type (IV), metaphysis-epiphysis distal end phalanx type (V) and bone joint type (VI)^7^. Among these, metaphysis change was a sensitive indicator and is easy to repair. The distal end phalanx change was a specific indicator but is hard to repair.

In practice, KBD monitoring is targeted for children aged 7–12 years old^8^. Because of the control of KBD, X-ray changes of typical KBD are hard to find, and children with other non-KBD abnormal X-ray signs are often misdiagnosed with KBD^9,10^. In this study, we identified some common confusing radiographic changes of KBD so as to exclude interferences and accurately identify positive X-ray signs of KBD in children.

## Results

We collected 3,193 valid X-ray images, including 1,399 images of Han children in KBD endemic areas (656 in Honggang District and 743 in Baiquan County) in Heilongjiang, 537 x-ray images of Tibetan children (all from Xaitongmoin County, a KBD endemic area of Tibet), and 1,257 images from non-KBD endemic areas (478 in Wulanchabu, Inner Mongolia, 358 in Ying County, Shanxi, and 421 in Taibai County, Shannxi), as shown in Table 1.

**Table 1:** The basic information of the regions and participants.

Areas	Ethnicities	Provinces	Counties (cities)	Gender			Age (years)					
				Total	Boys	Girls	7	8	9	10	11	12
KBD areas	Han	Heilongjiang	Honggang	656	337	319	56	137	135	122	123	83
			Baiquan	743	364	379	101	129	105	147	152	109
	Tibetan	Tibet	Xaitongmoin	537	296	241	63	58	120	62	128	106
Non-KBD areas	Han	Inner Mongolia	Wulanchabu	478	235	243	57	70	63	74	107	107
		Shanxi	Ying	358	157	201	31	74	77	70	65	41
		Shaanxi	Taibai	421	242	179	56	69	94	82	59	61
Total				3193	1631	1562	364	537	594	557	634	507

To compare the detection rates for all types of hand X-ray signs in children between KBD and non-KBD areas (or Han and Tibetan), the influences of gender difference needed to be controlled. Therefore, regions with similar gender composition were chosen for analysis.

Simultaneously, due to different age distributions, age-standardized detection rates were compared between KBD and non-KBD areas (or Han and Tibetan).

Baiquan County (KBD area) in Heilongjiang and Wulanchabu City (non-KBD area) in Inner Mongolia (gender difference was not statistically significant, χ^2^ = 0.003, p = 0.953) were selected for comparison of detection rates in KBD and non-KBD areas. Honggang District (Han) and Xaitongmoin County (Tibetan) (gender difference was not statistically significant, χ^2^ = 1.666, p = 0.197) were chosen for comparison of racial differences.

### Basic X-ray imaging results

No cases of KBD were found in KBD areas. Eleven KBD X-ray positive images were found. Six KBD-positive X-ray images were found in the non-KBD areas. All the KBD-positive X-ray variations were of the metaphysis type.

**Figure 1:**
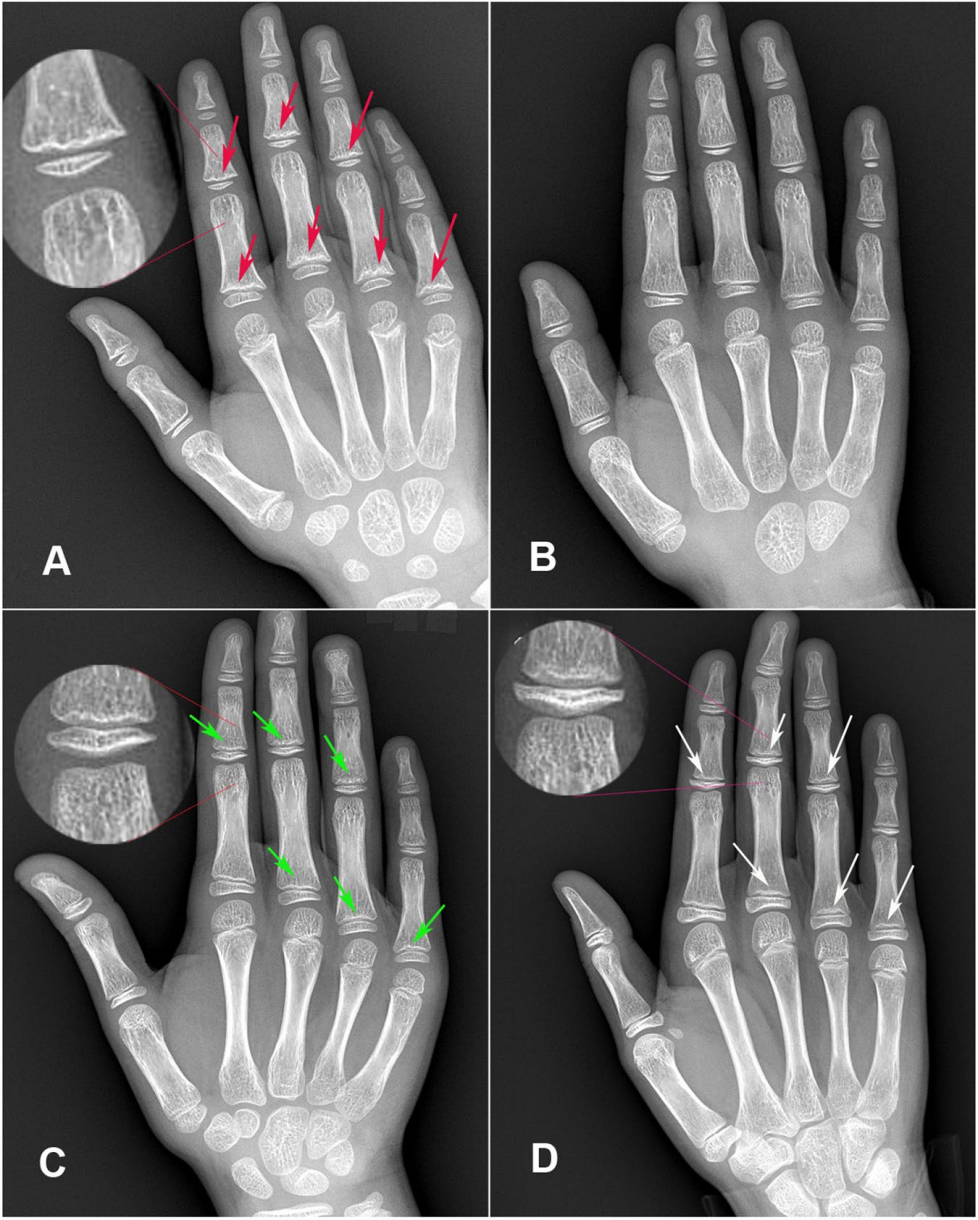
(**A**) Is a radiological image of the right hand with KBD-positive X-ray signs in an 8-year-old boy: some metaphyseal hardening signs, waviness or serration changes and small depressions (red arrows) are found in the zones of provisional metaphyseal calcification in the fingers (including index finger, middle finger and ring finger). (**B**) Is a radiological image of the right hand of a 7-year-old healthy boy: all metaphyses, epiphyses and diaphyses were glabrate, uniform, sinuous and non-destructive. The carpals were not completely formed. (**C**) Is a radiological image of the right hand of an 11-year-old girl with CRME in the equal-diameter period: the diameter of metaphysis and epiphysis are almost equal. Although it belongs to the normal development periods before complete closure of metaphysis-epiphysis (CCME), its imaging signs are similar to KBD-positive X-ray signs. Index finger, middle finger, ring finger and little finger show metaphyses serrated, wavy (green arrows). (**D**) Is a radiological image of the right hand of a 10-year-old girl with CRME in the ultra-diameter period: The diameter of epiphysis is longer than that of metaphysis. It occurs after the equal-diameter period normally and the features are similar to KBD-positive X-ray signs. Some metaphyseal waviness, serration and irregularly depressions are found in many fingers (white arrows).

**Figure 2:**
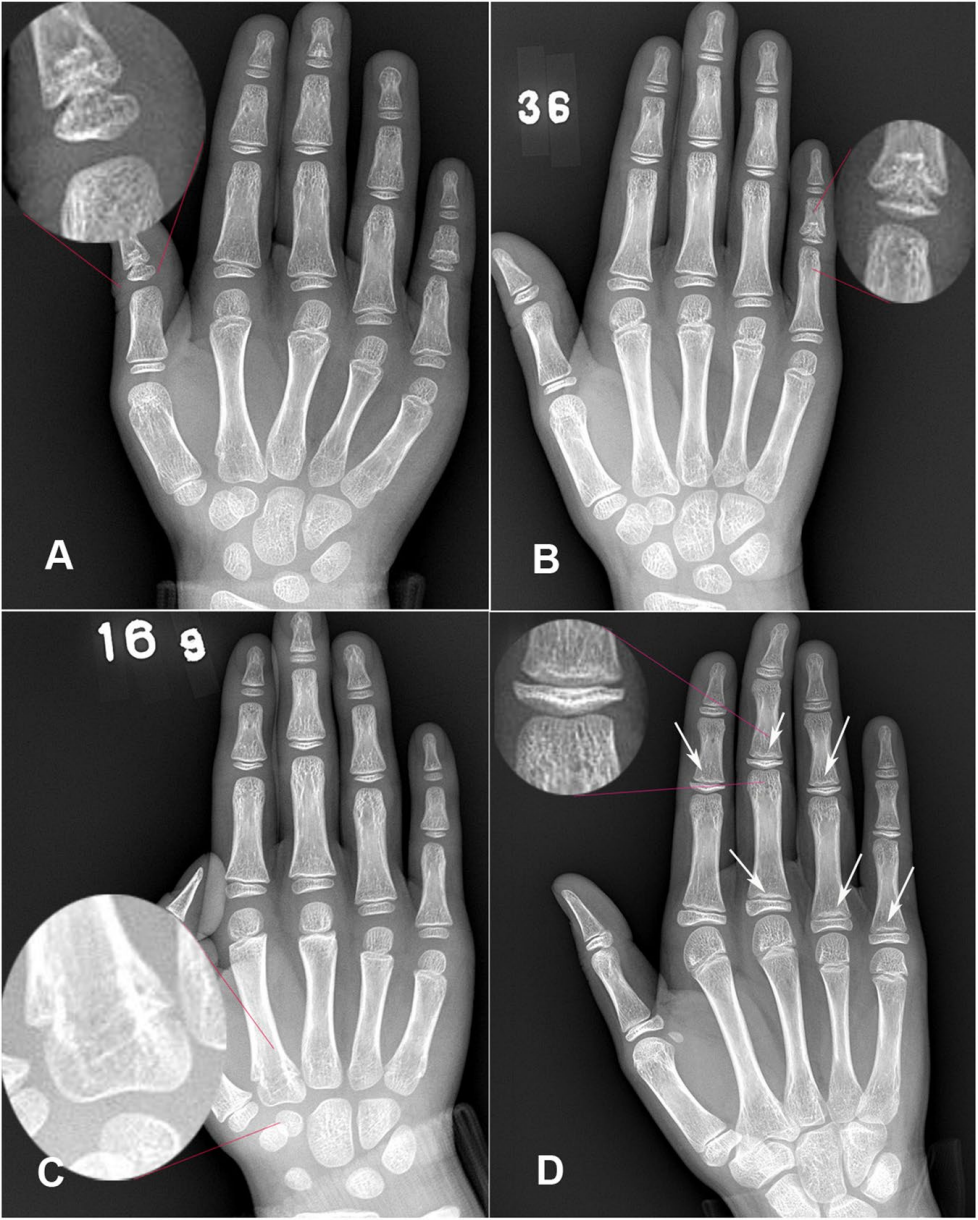
(**A**) Is an X-ray image of the right hand of a 7-year-old girl with changes of the thumb: cone-shaped epiphysis is embedding the metaphysis of the thumb singly. (**B**) Is a radiological image of the right hand of an 8-year-old girl with variation in the little finger: cone-shaped epiphysis was embedding metaphysis badly in middle phalanx of little finger. All the other parts were normal. (**C**) Is a radiological image of the right hand of an 8-year-old boy with the second metacarpal-phalangeal variation: Two epiphyses are connected to the second metacarpal. All the other parts are normal. (**D**) Is a radiological image of the right hand of a 10-year-old girl with cystic changes: cystic changes in index finger, middle finger, ring finger and little finger (yellow arrows).

In addition to the KBD-positive X-ray signs shown in Fig. 1A and healthy X-ray films shown in Fig. 1B, there were 5 types of abnormal variations in X-ray images. The survey revealed high incidence of closure reaction of metaphysis-epiphysis (CRME), including changes in the partial equal-diameter period (the period when epiphyseal diameter is equal to that of the metaphysis, as shown in Fig. 1C) and changes in most of ultra-diameter period (the period when the epiphyseal diameter is longer than the metaphyseal, as shown in Fig. 1D). The other variations of the phalanges included variations in the phalanx of the thumb (especially cone-shaped epiphysis, as shown in Fig. 2A), variations in median phalanx of the little finger (including the cone-shaped epiphysis, premature closure, short deformity, diaphysis change, as shown in Fig. 2B), the second metacarpal-phalangeal variation (Fig. 2C), and cystic changes (Fig. 2D). The main types of hand X-ray signs are shown in Table 2.

**Table 2:** Main types of hand X-ray signs in diagnosis of KBD in children.

Areas	Ethni-cities	Provinces	Counties (cities)	Simple sizes	KBD positive X-ray signs		CRME			Variations in thumb		Variations in little finger		The second metacarpal phalangeal variation		Cystic changes	
					Number of children	Detection rates (%)	Number of children	Detection rates (%)	Onset age (girl/boy)	Number of children	Detection rates (%)	Number of children	Detection rates (%)	Number of children	Detection rate (%)	Number of children	Detection rate (%)
KBD areas	Han	Heilong-jiang	Hong-gang	656	4	0.61	135	20.58	9/10	1	0.15	82	12.50	4	0.61	75	11.43
			Baiquan	743	6	0.81	137	18.44	10/11	3	0.40	65	8.75	0	0.00	14	1.88
	Tibetan	Tibet	Xaiton-gmoin	537	1	0.19	37	6.89	11/11	1	0.19	24	4.47	0	0.00	1	0.19
Non-KBD areas	Han	Inner Mongolia	Wulan-chabu	478	2	0.42	88	18.41	10/12	1	0.21	53	11.09	0	0.00	16	3.35
		Shanxi	Ying	358	2	0.56	33	9.22	11/12	0	0.00	31	8.66	0	0.00	5	1.40
		Shaanxi	Taibai	421	2	0.48	26	6.18	10/11	1	0.24	29	6.89	0	0.00	12	2.85
Total				3193	17	0.53	456	14.28		7	0.22	284	8.89	4	0.13	123	3.85

No cases of KBD in children were found in KBD areas. Few children (0.53%) with KBD-positive X-ray variations in hand bones were found (all were metaphysis changes). The confusing change types in KBD diagnosis included CRME, thumb variation, little finger variation, the second metacarpal phalangeal variation and cystic change. CRME (14.28%) was most likely to interfere with radiological diagnosis of KBD. The onset age of CRME in girls (9) was earlier than that in boys (10). Onset occurred earlier in Han children (9) than in Tibetan children (11) and occurred earlier in KBD area children (9) compared with non-endemic area children (10). The other four types (detection rates were 0.22%, 8.89%, 0.13%, 3.85%, respectively) could be distinguished from KBD by imaging alone.

Although 5 types of X-ray abnormal variations were detected, during the reviewing process, CRME was the major confusing change. Therefore, its epidemiological characteristics were analysed.

### Comparison of detection rates of CRME between Han and Tibetan children in the KBD endemic areas

Honggang District (Han) and Xaitongmoin County (Tibetan) (gender difference was not statistically significant, χ^2^ = 1.666, p = 0.197) were chosen for racial comparison of detection rates of closure reaction of metaphysis-epiphysis (Table 3). Because of the different age distributions, age-standardized detection rates were applied and compared between Han and Tibetan (Table 4).

**Table 3:** Detection rates of CRME in 7- to 12-year-old Han and Tibetan children.

Age (years)	Han (Honggang)						Tibetan (Xaitongmoin)					
	Boys			Girls			Boys			Girls		
	Number of investigated children	Number of abnormal children	Detection rates (%)	Number of investigated children	Number of abnormal children	Detection rates (%)	Number of investigated children	Number of abnormal children	Detection rates (%)	Number of investigated children	Number of abnormal children	Detection rates (%)
7	31	0	0	25	0	0	36	0	0	27	0	0
8	67	0	0	70	0	0	28	0	0	30	0	0
9	68	0	0	67	11	16.42	69	0	0	51	0	0
10	58	2	3.45	64	19	29.69	33	0	0	29	0	0
11	64	11	17.19	59	45	76.27	71	1	1.41	57	9	15.79
12	49	19	38.78	34	28	82.35	59	2	3.39	47	25	53.19
Total	337	32	9.50	319	103	32.29	296	3	1.01	241	34	14.11

**Table 4:** Age-standardized detection rates of closure reaction of metaphysis-epiphysis in 7- to 12-year-old Han and Tibetan children.

Age (years)	Standard number of children	Honggang (Han)				Xaitongmoin (Tibetan)			
		Boys		Girls		Boys		Girls	
		Detection rates (%)	Expected number of abnormal children	Detection rates (%)	Expected number of abnormal children	Detection rates (%)	Expected number of abnormal children	Detection rates (%)	Expected number of abnormal children
7	119	0	0	0	0	0	0	0	0
8	195	0	0	0	0	0	0	0	0
9	255	0	0	16.42	41.87	0	0	0	0
10	184	3.45	6.35	29.69	54.63	0	0	0	0
11	251	17.19	43.15	76.27	191.44	1.41	3.54	15.79	39.63
12	189	38.78	73.29	82.35	155.64	3.39	6.41	53.19	100.53
Total	1193	10.30*	122.79	37.18*	443.58	0.83*	9.95	11.75*	140.16

Note: * indicates age-standardized detection rate.

Table 4 showed that CRME was related to age, gender and ethnicity. The age-standardized detection rate of Han girls (37.18%) was significantly higher than that of boys (10.30%). Onset of CRME in Han girls (9 years old) was earlier than in boys (10 years old). There are similarities in Tibetan children. The age-standardized detection rate of CRME was 47.48% (10.30% plus 37.18%) in Han, higher than that of Tibetan (12.85%, that is, 0.83% plus 11.75%). CRME in Han children occurred earlier than in Tibetan children. Onset of CRME in Tibetan boys was at 11 years old, compared to age 10 for Han boys. Onset of CRME for Tibetan girls was at 11 years old, compared with age 9 for Han girls.

### Comparison of CRME detection rates in children in Han in KBD area with that in non-KBD area

Baiquan County (KBD area) in Heilongjiang and Wulanchabu City (non-KBD area) in Inner Mongolia (gender difference was not statistically significant, χ^2^ = 0.003, p = 0.953) were selected for comparison of detection rates of CRME between KBD and non-KBD areas (Table 5). Due to the different age distributions, age-standardized detection rates were applied and compared between KBD and non-KBD areas (Table 6).

**Table 5:** Detection rates of CRME in 7- to 12-year-old children in endemic and non-KBD areas.

Age (year)	Baiquan (KBD area)						Non-KBD area (Wulanchabu)					
	Boys			Girls			Boys			Girls		
	Number of investigated children	Number of abnormal children	Detection rates (%)	Number of investigated children	Number of abnormal children	Detection rate (%)	Number of investigated children	Number of abnormal children	Detection rates (%)	Number of investigated children	Number of abnormal children	Detection rates (%)
7	46	0	0	55	0	0	31	0	0	26	0	0
8	65	0	0	64	0	0	37	0	0	33	0	0
9	53	0	0	52	0	0	26	0	0	37	0	0
10	80	0	0	67	3	4.48	34	0	0	40	3	7.5
11	67	3	4.48	85	62	72.94	49	0	0	58	28	48.28
12	53	18	33.96	56	51	91.07	58	16	27.59	49	41	83.67
Total	364	21	5.77	379	116	30.61	235	16	6.81	243	72	29.63

**Table 6:** Standardized detection rates of CRME in 7–12-year-old children in KBD and non-KBD areas.

Age (year)	Standard number of children	Baiquan (KBD area)				Wulanchabu (Non-KBD area)			
		Boys		Girls		Boys		Girls	
		Detection rates (%)	Expected number of abnormal children	Detection rates (%)	Expected number of abnormal children	Detection rates (%)	Expected number of abnormal children	Detection rates (%)	Expected number of abnormal children
7	158	0	0	0	0	0	0	0	0
8	199	0	0	0	0	0	0	0	0
9	168	0	0	0	0	0	0	0	0
10	221	0	0	4.48	11.60	0	0	7.5	16.58
11	259	4.48	11.60	72.94	188.91	0	0	48.28	125.05
12	216	33.96	73.35	91.07	196.71	27.59	59.59	83.67	180.73
Total	1221	6.96*	84.95	32.53*	397.22	4.88*	59.59	26.40*	322.36

Note: * indicates age-standardized detection rate.

Table 6 showed that the CRME was related to age, gender and area. The age-standardized detection rate of girls (32.53%) was significantly higher than that of boys (6.96%) in the KBD area. Onset of CRME occurred earlier in girls (10 years old) than in boys (11 years old) in the KBD area. There are similarities in the non-KBD area. The age-standardized detection rate of CRME was 39.49% (6.96% plus 32.53%) in the KBD area, higher than that of the non-KBD area (31.28%, that is, 4.88% plus 26.40%). Onset of CRME occurred earlier in boys in the KBD area (11 years old) compared with boys in the non-KBD area (12 years old).

## Discussion

KBD is an endemic disease characterized by multiple chronic osteoarthropathies whose pathological changes are degeneration, necrosis and secondary osteoarthritis in articular cartilage of the extremities. Severe KBD may manifest as short height, deformity and disability for life. The aetiology and pathology of KBD remain under investigation. KBD is distributed from Sichuan-Tibet region to the northeast of China and spreads to Eastern Siberia in Russia and the northern mountainous area of North Korea^11^. KBD patients are not found in other regions of the world. In China, KBD is diagnosed in 21,436 villages of 378 counties in 13 provinces, affecting approximately 21.97 million residents.

In “Endemic Disease”, the change rate of children’s metacarpal phalanges was used as a sensitive indicator while screening for KBD. The prevalence of KBD has been effectively controlled coincident with the rapid development of the economy, society and quality of life, as well as the implementation of comprehensive preventive measures^12^. From the surveillance data of 13 provinces in 2014, the clinical detectable rate of children with KBD was 0.01%, and the X-ray positive detectable rate was 0.17%^13^. The survey showed that the detection rate of KBD-positive X-ray change in endemic areas (i.e., the severe KBD disease areas of many years ago) was lower than the standard value for eliminating KBD^8^ (X-ray positive detectable rate ≤3%) (Honggang 0.61%, Baiquan 0.81%, Xaitongmoin 0.19%). This finding suggests that KBD has been effectively controlled in these areas. This control is attributed to the contributions of domestic and foreign organizations, as well as prevention and control efforts.

To prevent the re-emergence of KBD, monitoring is essential. Hand X-ray examination of children has been the most important measurement in KBD screening. However, there is high rate of misdiagnosis due to many other X-ray changes that are easily confused with KBD changes^14^. To reduce this interference, it is necessary to improve diagnostic accuracy.

According to “Diagnosis of Kashin-Beck Disease” (WS/T 207-2010), the radiographic features of KBD are primary symmetrical sclerosis, depression, deformation of the early calcification zone of the diaphysis or metaphysis (may without KBD clinical characteristics). KBD clinical characteristics are based on more than 6 months of contact history, symmetric multiple swelling or deformity of finger (or toe) joints, radiographic features of KBD, and exclusion of other related diseases.

In this study, many variations of hand bones were found in children, including changes of CRME, variations in the phalanx of the thumb, variations in the median phalanx of little finger, the second metacarpal-phalangeal variation, and cystic changes. In radiology, some also presented with multiple symmetrical sclerosis, depression, and deformation of the early calcification zone of the metaphysis or diaphysis. These radiographic abnormalities revealed secondary repair and remodelling of the adjacent bone and cartilage of the metaphyses and epiphyses in response to cartilaginous necrosis^15^. Of all of these abnormalities, CRME was the most commonly confused with KBD, leading to misdiagnosis as a radiologically positive KBD^16^.

Previous studies defined six positive X-ray changes, including metaphysis type (I), distal end phalanx type (II), metaphysis-epiphysis type (III), metaphysis distal end phalanx type (IV), metaphysis-epiphysis distal end phalanx type (V) and bone joint type (VI), based on the sites of the child’s hand bones. Metaphyseal change is the earliest and mildest KBD type and is also easiest to repair^17^. Although metaphysis type is a sensitive indicator, it is not a specific indicator (distal end phalanx changes are specific indicators). As the metaphyses of the phalanges are highly metabolized and sensitive to the external environment, a number of other factors can also cause metaphyseal changes in non-KBD areas, including traumatic arthritis, congenital and myxoedema, but the incidence is low. We found metaphyseal changes in non-KBD cases (Wulanchabu 0.42%, Ying 0.56%, Taibai 0.48%).

In this study, all the detection variations of KBD-positive X-ray were metaphysis changes that manifested as wave-like metaphyses, jagged changes, were accompanied by widening, hardening or small depression, or were symmetrical multiple. The metaphyseal anterior calcification bands of median segments and coxal bones in the index finger, middle finger, ring finger were serrated and wavy.

According to KBD diagnostic criteria, KBD symptoms are simultaneous changes in multiple phalanges. Simple thumb variations or little finger variations and cystic changes were not characteristic X-ray changes of KBD. These X-ray signs should be identified in KBD diagnosis.

Previous studies reported typical signs of X-rays of KBD^7^. However, they did not involve the imaging features of other changes easily confused with KBD, although the incidence of these changes is relatively low. In the previous era of epidemic KBD, the false positive rate caused by these changes was low, but now, there are fewer new cases of KBD. Very few confounding factors may lead to severe inaccuracies in KBD monitoring data. This study showed that of these disturbing changes, the most common was CRME.

CRME belongs to the range of normal development before complete closure of the metaphysis-epiphysis (CCME). CRME occurs mainly in the ultra-diameter period and less often in the equal-diameter period (both of them are peculiar changes in the adolescent period). In both periods, the metabolism of the phalanges is relatively vigorous. Metaphysis was prone to wavy or saw tooth-like changes, or accompanied by a small depression, or with early calcification and hardened widening. The median phalanx of the little finger is also prone to the presence of conical epiphysis embedded in metaphysis before CCME^18^. These changes usually occur in each metacarpal phalanx with symmetrical distribution. Children with CRME should be excluded from KBD diagnosis as soon as possible, as clearly defined in “Diagnostic criteria of KBD”. Although CRME is difficult to distinguish from KBD-positive X-ray changes, it can be excluded in epidemiology.

Early research reported that the age of onset of CCME in girls is earlier than that of boys^19^. The present study showed that the age of CRME in girls is earlier than that of boys, which is in line with physiological logic.

The study showed racial differences in terms of age of CRME. The age-standardized detection rates of the CRME of Han are higher than that of Tibetan. Han CRME occurred earlier than the Tibetan.

The study also showed regional differences in the age of CRME. The age-standardized detection rate of CRME in KBD areas was higher than that of non-KBD areas. Initial CRME in boys in KBD areas was earlier than that of non-KBD area. Previous studies reported that metaphysis-epiphysis changes in children were related to age, gender and nutritional status^20–22^, but the reaction before the complete closure has not been reported, and its influence in KBD monitoring has not been analysed.

The study sites in Baiquan County (KBD area) in Heilongjiang and Wulanchabu City (non-KBD area) in Inner Mongolia had similar economic levels (backward rural areas, after controlling for the effects of economic and nutritional status). Therefore, we speculated that this may be related to other factors that promote the early closure of the bones. Although the aetiology of KBD is not clear, KBD can lead to premature closure of phalanges and may limit the growth of children. Some severe cases may have short fingers, short legs, and even short stature. The results of this study suggested that after considering the factors of age, gender and ethnicity, premature CRME may be related to the risk factors for KBD.

We found some hints for KBD monitoring in the future. To reduce the workload and cost of monitoring, as well as to reduce the rates of misdiagnosis, we suggest that the monitored age range should be adjusted to 7–11 years old (investigate only boys in 11-year-olds) for Han children and should be at 7–12 years old (investigate only boys in 12-year-olds) for Tibetan children.

In conclusion, KBD has been effectively controlled in China. This study contemplated five types of metacarpal and phalangeal variations which were confused in KBD-positive X-ray diagnosis. The one that should attract most attention was CRME, which was associated with age, gender, and ethnic differences. To reduce the misdiagnosis rates in KBD surveillance, the age range should be adjusted to 7–11 years old (investigate only boys in 11-year-olds) in Han children and should remain at 7–12 years old (investigate only boys in 12-year-olds) for Tibetan children. In addition, the fact that the onset age of CRME in children is earlier in endemic areas than in non-endemic areas may be partially due to the influence of KBD risk factors, which lead to the obstruction of bone development and to premature CRME.

## Methods

### Ethical standards disclosure

This study was conducted according to the guidelines established by the Declaration of Helsinki, and all procedures involving human subjects/patients were approved by the Chinese Center for Disease Control and Prevention (CDC). Verbal informed consent was obtained from the parents of the children.

### Investigated regions and participants

The study was performed from October 2009 to April 2015 using typical sampling methods. The KBD endemic areas were Honggang District in Heilongjiang Province (data collected in 2014), Baiquan County in Heilongjiang Province (data collected in 2015), and Xaitongmoin County (Tibetan neighbourhoods) in Tibet Autonomous Region (data collected in 2014). Non-KBD areas were Wulanchabu city in Inner Mongolia Autonomous Region (data collected in 2010), Ying County in Shanxi (data collected in 2009) and Taibai County in Shaanxi (data collected in 2009). Approximately one to three towns in each county or district were selected. Then, 3~5 villages in each town were selected. In these villages, all 7- to 12-year-old children were selected as subjects. Finally, 3193 children were enrolled in this study.

### Instruments and methods

The high-frequency portable digital medical diagnostic X-ray image DR system (Beijing Langsafe Imaging Technology Co., Ltd) was used to obtain X-ray images. Subjects were asked to close their fingers together, palm down, flat on the screen, keeping the wrist with the hand in a straight line for optimum image acquisition.

KBD was diagnosed according to the Health Care Professional Standard “Diagnosis of Kashin-Beck Disease” (WS/T 207-2010)^23^. Clinic patients with KBD must have life history of more than six months living in KBD endemic areas. Simultaneously, hand X-ray images must be KBD-positive.

### Imaging diagnosis

The X-ray images were read by three KBD experts. The results of the diagnosis must be approved by two or more experts. Inconsistent results of hand images were diagnosed again as before. Then, the results of the second diagnosis were used for subsequent statistical analysis. Finally, we analysed the types and detection rates of metacarpal and phalangeal changes.

### Statistical Analysis

SPSS version 20 (IBM, Armonk, NY) software for statistical analysis. The χ^2^ test was applied for gender comparison (α = 0.05). The standardized rates were applied to eliminate the influence of age on the detection rate of abnormalities of the metacarpals and phalanges.
